# Modulation and Reprogramming of Adipose Tissue Macrophages in Obesity

**DOI:** 10.3390/biom16020339

**Published:** 2026-02-23

**Authors:** Yan Liu, Xiaoying Huang, Linfeng Sang, Yang Zhang, Jiajie Cao, Qin Kong

**Affiliations:** 1 Department of Pharmacology, School of Pharmacy, Anhui Medical University, Hefei 230032, China; 2445010206@stu.ahmu.edu.cn (Y.L.); 2213051113@stu.ahmu.edu.cn (X.H.); 2113051017@stu.ahmu.edu.cn (L.S.); 2345010209@stu.ahmu.edu.cn (Y.Z.); 2 School of Nursing, Anhui Medical University, Hefei 230032, China; 2413070056@stu.ahmu.edu.cn; 3 Anhui Provincial Institute of Translational Medicine, Anhui Medical University, Hefei 230032, China

**Keywords:** obesity, adipose tissue, macrophages, metabolic reprogramming

## Abstract

Chronic inflammation associated with obesity drives metabolic dysfunctions and induces immune cell maintenance. Adipose tissue macrophages (ATMs), the predominant immune population within adipose depots, exhibit substantial heterogeneity and serve as central pathological mediators in obesity-induced adipose inflammation and metabolic dysregulation. In this review, we highlight the reprogramming of ATMs’ metabolic pathways, including glucose and lipid homeostasis associated with obesity, with a focus on chronic metabolic inflammation. Additionally, we discuss underlying mechanisms supporting ATMs remodeling in obesity, including transcriptional control and epigenetic regulation.

## 1. Introduction

Obesity represents a pathological state characterized by excessive adipose tissue expansion, driven by disrupted energy homeostasis due to caloric excess. This lipid deposition induces a chronic, low-grade inflammation that serves as a primary driver for the pathogenesis of type 2 diabetes (T2D), metabolic dysfunction-associated fatty liver disease (MAFLD), and cardiovascular diseases [1]. As a crucial energy reservoir and a dynamic endocrine organ, adipose tissue exhibits high plasticity and undergoes profound remodeling under obese conditions: adipocyte hypertrophy-induced hypoxia, endoplasmic reticulum (ER) stress, and the inflammatory microenvironment formation [2,3]. These factors collectively promote the recruitment of immune cells, especially ATMs, thereby establishing a self-perpetuating metabolic–inflammatory cycle.

ATMs are the primary immune cell population associated with obesity. Recent single-cell transcriptomic analyses have redefined their functional heterogeneity, moving beyond the traditional M1/M2 paradigm. ATM subtypes are now classified by spatial localization, molecular signatures, and functional properties. Spatially distinct ATMs exhibit significant functional specialization. Yu defined three distinct ATM populations, characterized by the expression of CD209b and LYVE1 (CD209b^+^LYVE1^+^), based on subtissular localization and molecular signatures. Selective genetic depletion of septal ATMs enhanced thermogenesis, white adipose tissue (WAT) beiging, and protection from high-fat diet (HFD)-induced obesity, along with improved glucose tolerance and insulin sensitivity [4]. Pirzgalska identified sympathetic neuron-associated macrophages (SAMs) within adipose tissue that adopt a pro-inflammatory phenotype via sympathetic nervous system activation. SAMs express the norepinephrine transporter solute carrier family 6 member 2 (SLC6A2) and monoamine oxidase A, which collectively facilitate neurotransmitter uptake and degradation. Genetic ablation of Slc6a2 in these macrophages markedly enhances lipolysis and thermogenesis [5]. Furthermore, vascular-associated macrophages (VAMs)-, lipid-associated macrophages (LAMs)-, and crown-like structures (CLSs)-associated ATMs play unique roles in maintaining the homeostasis of the adipose tissue microenvironment [6,7,8,9]. Mechanistically, β2-adrenergic receptor signaling induces specific ATMs subpopulations, designated cholinergic adipose macrophages (ChAMs), to secrete acetylcholine and thereby regulate subcutaneous adipose thermogenesis [10]. ATM populations are broadly categorized as tissue-resident (including perivascular (PVM) and non-perivascular macrophages (NPVM)) or recruited. Tissue-resident ATMs are essential for maintaining tissue homeostasis, whereas recruited subsets largely contribute to inflammatory responses [11,12].

ATMs exhibit dynamic phenotypic plasticity during the progression of metabolic diseases, encompassing the traditional M1/M2 states, oxidized states, and metabolically activated states, which is an emerging concept [13,14]. Characterizing these specialized ATM subsets elucidates key mechanisms in obesity and highlights novel therapeutic opportunities. This emerging understanding may advance strategies to disrupt metabolic inflammation, potentially leading to improved prevention and treatment of obesity and its complications.

ATMs sustain adipose tissue homeostasis and drive metabolic inflammation. Given the extensive functional heterogeneity and phenotypic plasticity of ATMs, defining the precise mechanisms driving their reprogramming in obesity is essential. Evidence indicates that obesity triggers dynamic remodeling of carbohydrate, lipid, and lactate metabolism in ATMs, establishing distinct metabolic adaptations. This reprogramming not only modulates inflammatory responses both systemically and within adipose tissue but also impairs systemic energy homeostasis. In this review, we provide an overview of the subset of ATMs, metabolic reprogramming of ATMs in chronic metabolic diseases, the underlying mechanisms, and our current understanding.

## 2. Overview of Adipose Tissue Macrophages (ATMS) and Metabolic Dysfunction

White adipose tissue (WAT) possesses a remarkable regulatory capacity, enabling it to adjust its size and function in response to diverse internal and external cues, such as biomechanical forces and nutritional status. White adipocytes play a central role in systemic energy homeostasis through their metabolism of critical substrates like glucose and lipids. The dysregulation of these metabolic pathways is closely linked to metabolic disorders and adipose tissue dysfunction. In lean adipose tissue, macrophages maintain homeostasis through efferocytosis, phagocytosis, and the secretion of anti-inflammatory cytokines. 

In this state, ATMs primarily originate from the local proliferation of yolk-sac progenitors and are distributed along vasculature or between adipocytes. In contrast, during obesity, the proportion of macrophages increases significantly, indicating their active role in adipose tissue. This expansion stems primarily from the enhanced recruitment and proliferation of Ly6C^+^ and CCR2^+^ monocytes [15]. These ATMs predominantly localize around dead adipocytes, forming crown-like structures (CLSs). In brief, ATM heterogeneity evolves significantly as obesity progresses, shifting from an anti-inflammatory homeostasis toward a diverse, pro-inflammatory state that drives metabolic dysfunction. Macrophage activity is governed by its sensitivity to the local tissue microenvironment and the consequent immunometabolic adaptations. A key determinant of macrophage state is its metabolism, specifically the flux through various pathways, including glucose, fatty acid, ketone, and amino acid metabolism. Fundamentally, macrophages are cells for which sustaining metabolism is a primary task. This review summarizes the metabolic shifts in oxidizable substrates (glucose, lipids, and ketone) within macrophages and examines how these alterations influence macrophage polarization and inflammation in obesity.

### 2.1. ATMs

**Figure 1:**
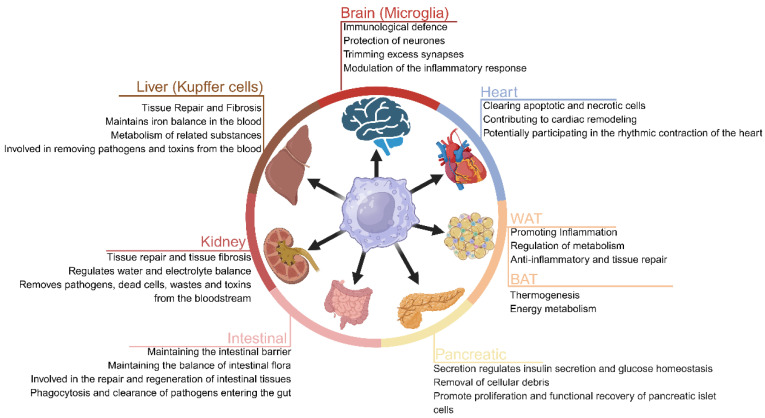
Tissue-specific roles of macrophages in metabolism. Macrophages are widely distributed in metabolic organs and play multiple roles. They also participate in the process of tissue repair while protecting organs from excessive damage. This figure highlights their distinct functions in different adipose depots: driving inflammation and remodeling in WAT, and modulating thermogenesis in BAT. WAT, White adipose tissue. BAT, Brown adipose tissue.

The heterogeneity of tissue macrophages is increasingly recognized, both in health and disease. The macrophage inherent functional properties enable them to maintain tissue homeostasis through phagocytic clearance and the regulation of tissue repair [11]. Macrophages are essential cells found in almost every organ, where they display remarkable plasticity and contribute to various physiological processes in a tissue-specific manner (Figure 1). 

In obesity, ectopic lipid accumulation triggers the expansion and functional reprogramming of ATMs. The failure of adipocytes to maintain metabolic homeostasis results in loss of lipid and glucose homeostasis, ultimately leading to chronic inflammation. ATMs display considerable heterogeneity, which is evident in their ontogeny and functional characteristics. ATMs ontogeny involves embryonic-derived, tissue-resident macrophages and bone-marrow-derived monocyte (BMDM) recruitment [16,17]. Tissue-resident macrophages are defined by their self-renewal capacity, long-term persistence independent of BMDM, and stable, specific integration with parenchymal cells, thereby constituting an intrinsic component of a given tissue [18,19]. The recruitment of BMDM precursors represents another significant source. Monocytes, differentiated from hematopoietic stem cells in the bone marrow, can be recruited to adipose tissue and subsequently differentiate into macrophages under specific conditions, such as inflammatory responses [20,21]. Notably, adipose resident macrophages in obese mice refrain from producing inflammatory mediators, a behavior contrasting sharply with BMDMs. These two macrophage subsets coexist in adipose tissue, with their relative proportions and functional phenotypes dynamically shifting across physiological and pathological states. During obesity or overnutrition, ATMs abundance increases substantially, comprising 40–50% of stromal cells in adipose tissue [21,22]. Obesity triggers a cascade of immunometabolic responses characterized by ATMs secretion of chemokines and pro-inflammatory mediators, including *Ccl2*, *Ii1b*, *Il6*, *Tnf*, and *Nox2*. These factors also drive the recruitment and proliferation of pro-inflammatory macrophages, thereby initiating inflammatory cascades, ectopic lipid deposition, and insulin resistance [23,24,25]. C-C motif chemokine receptor 2 (CCR2)-deficient mice exhibit protection against obesity-associated complications without increased body weight, indicating that CCR2 ablation improves metabolic function [26]. In contrast, evolutionarily conserved resident macrophages secrete growth factors Pvf3 or PDGFcc (the direct homolog of Pvf3), coupling energy intake to lipid storage. This pathway exacerbates obesity and metabolic dysregulation through mechanisms distinct from CCR2-dependent macrophage-mediated inflammation. CCR2-dependent BMDMs recruited to obese adipose tissue drive inflammation via tumor necrosis factor (TNF) production, whose elevation in the adipose tissue of obese mice was first reported 30 years ago, thereby inducing systemic insulin resistance and ectopic lipid deposition [19,27]. Chronic low-grade inflammation is now recognized as a key pathological feature of obesity and its associated complications, including type 2 diabetes (T2D) and cardiovascular disease. 

For decades, ATMs were predominantly classified within a M1/M2 framework. Pro-inflammatory M1 ATMs are associated with innate immunity and inflammatory responses, while M2 ATMs are linked to anti-inflammatory processes, tissue repair, and fibrosis. However, this M1/M2 paradigm, utilized for over twenty years, is now acknowledged to possess significant limitations and faces conflicting experimental observations. One reason for this is the overly simplistic nature of the model, despite numerous researchers having proposed subtypes within these categories [28].

### 2.2. Diversity of ATMs

In response to metabolic stress in the adipose tissue microenvironment, we have refined macrophage classification strategies to delineate their dynamic phenotypic shifts (Table 1). Ly6C^+^ monocytes derived from bone marrow represent the primary infiltrating monocyte subset tissues during chronic inflammation and obesity [29]. Recruited monocyte-derived Ly6C^+^ macrophages proliferate in obesity, where they drive key pathological processes by secreting inflammatory cytokines and promoting adipogenesis [30,31,32].

HFD- and obesity-driven factors enhance Ly6C^+^ monocyte infiltration into the adipose tissue, despite an initial increase in Ly6C^−^ monocytes during early HFD exposure. Interestingly, when BMDMs were treated with bone marrow adipocyte-conditioned medium (BMA-CM) derived from HFD-feeding mice, it was found that the BMA-CA induces phenotypic whitening of bone marrow adipocytes. This progress promotes the induction of Ly6C^+^ monocytes into the bone marrow, thereby contributing to obesity-associated chronic low-grade inflammation [32]. 

A novel macrophage subset characterized by the markers of CD9 and CD63 was identified in the adipose tissue of obese mice. Lineage tracing with membrane-spanning-4-domains subfamily-A member-3 cre (Ms4a3Cre) demonstrated that these CD9^+^CD63^+^ macrophages are derived from bone marrow monocytes. This subset is distinguished by its high lipids and robust exosome secretion. Furthermore, these macrophages exhibit enhanced lysosomal activity and upregulated expression of pro-inflammatory genes, including *Acp5*, *Ctss*, *Lamp2*, *Lipa*, *Ccl2*, *Il1a*, *Il18*, and *Tnf* [30]. Notably, in mice subjected to a HFD, CD9^+^ macrophages formed CLS surrounding adipocytes and were found to be co-localized with LAM regions [6,11]. In parallel, obesity-associated metabolic dysfunction has been shown to drive a mechanistically distinct macrophage activation state, termed metabolically activated macrophages (MMe) [42]. This activation can be recapitulated in vitro by treatment with a combination of glucose, insulin, and palmitic acid, with palmitate serving as a canonical inducer. MMe macrophages upregulate a suite of inflammatory mediators, including *Nox2*, *Il1b*, *Cd36*, *Plin2*, *Lamp1*, and *Lamp2*, whose expression levels are influenced by the metabolic microenvironment. Central to MMe-driven inflammation is Nox2, which also facilitates the lysosomal exocytosis of apoptotic adipocytes. Accordingly, Nox2-deficient mice exhibit protective metabolic benefits during the early phase of HFD feeding, such as reduced ATMs inflammation. In contrast, these initial improvements are offset during later stages by the accumulation of ATMs’ cell death. Despite the continued high expression of pro-inflammatory and lipid metabolism genes, macrophages facilitate the clearance of dead adipocytes through lysosomal exocytosis via Nox2 in later diet-induced obesity [14,41,42]. While sharing a capacity to secrete pro-inflammatory cytokines, MMe and classically activated macrophages are distinguished by their surface profiles. MMe macrophages are uniquely characterized by elevated expression of the lipid metabolism proteins ABCA1, CD36, and PLIN2, markers commonly linked to M2-like phenotypes.

Trem2 is a key lipid receptor on macrophages, predominantly localized to CLS, where it regulates essential processes, including inflammation, phagocytosis, and metabolism [35]. Trem2^+^ macrophages as key players in sensing and clearing extracellular lipids, thereby curbing adipocyte hypertrophy and protecting systemic metabolic homeostasis. Trem2 deficiency disrupts macrophage recruitment, exacerbating adipocyte enlargement, hypercholesterolemia, inflammation, and glucose intolerance [6]. Separately, in the context of transarterial chemoembolization (TACE), Trem2^+^ tumor-associated macrophages (TAMs) contribute to immunosuppression within the tumor microenvironment. These TAMs show reduced Cxcl9 expression-a chemokine critical for antitumor immune cell recruitment and are associated with upregulated PD-L1 in endothelial cells. This elevation in PD-L1 promotes Galectin-1 secretion, further inhibiting CD8^+^ T cell infiltration and correlating with poorer clinical outcomes [43]. The iron-rich (MFe^hi^) ATM subset was initially identified as tissue-resident macrophages with an M2-like profile. Their presence highlights the critical role of iron homeostasis in adipose tissue, suggesting the involvement of macrophage-dependent regulatory pathways [39]. Recently, Luo et al. described a distinct inflammatory and metabolically activated macrophage (iMAM) subset characterized by high-protein disulfide-isomerase A3 (PDIA3) expression. These PDIA3^hi^ macrophages represent a pathogenic ATMs subpopulation in which PDIA3 supports their migratory and pro-inflammatory functions [3]. 

## 3. Multiple Roles of Macrophages in the Adipose Tissue Microenvironment

The adipose tissue microenvironment comprises the biochemical and physical conditions that support and modulate the behavior of resident cells, including adipocytes (both preadipocytes and mature adipocytes), macrophages, endothelial cells, and fibroblasts [44]. This microenvironment is shaped by diverse factors, such as mechanical forces, which influence cellular development, homeostasis, and repair through mechano-transduction. For example, immune cells sense mechanical stress from their surroundings and convert them into biochemical signals that activate intracellular cascades. As innate immune cells, macrophages detect substrate stiffness via mechanosensitive receptors, thereby promoting efficient phagocytosis [45]. Cellular metabolism—which underlies energy production and biomolecule synthesis—serves as the foundational support for all cellular functions. Accordingly, shifts in systemic metabolism reshape the adipose tissue microenvironment, driving adaptive responses [46]. During obesity, for instance, physical changes (hypoxia and ECM) and biochemical alterations, such as chronic inflammation within adipose tissue, prompt profound cellular reprogramming. ATMs relocate and shift their polarization state from an anti-inflammatory phenotype to a pro-inflammatory state. ATM-derived IL-10 confers protection against TNF-α-induced insulin resistance in lean mice adipocytes. By contrast, diet-induced obesity promotes a transition from M2-like ATMs toward a dominant M1-like pro-inflammatory population, which exacerbates insulin resistance [13,47]. 

**Figure 2:**
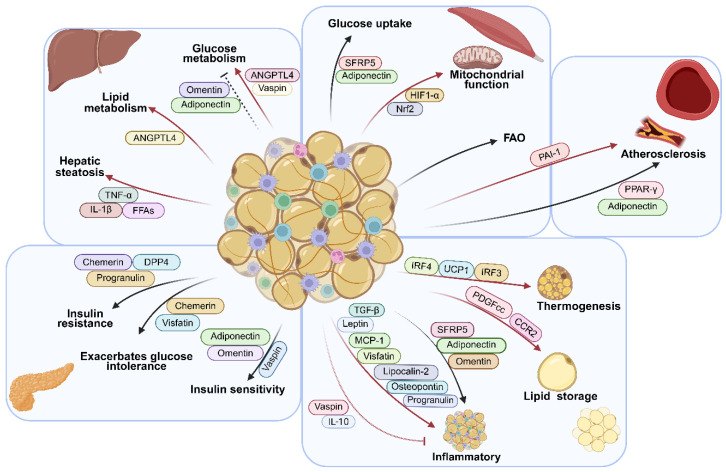
Adipose tissue cross-talk with other tissues via adipokines. Adipose tissue acts as a key endocrine organ producing adipokines and exert their effects on distant tissues. Systemic regulation of major adipokines in energy metabolism. As a key endocrine organ, adipose tissue secretes adipokines that exert systemic effects on insulin sensitivity, glucose and lipid homeostasis, as well as immune and cardiovascular functions. In this figure, red lines indicate increases (up-regulation), while black lines denote decreases (down-regulation).

ATMs play key roles in both immune surveillance and the regulation of metabolic and inflammatory homeostasis. Through the secretion of diverse cytokines and chemokines, these cells exert profound effects on adipose tissue function and systemic metabolism. Under physiological conditions, macrophages produce anti-inflammatory factors, such as IL-10 and TGF-β, which contribute to tissue repair and inflammatory balance. However, in obesity, elevated TGF-β secretion promotes adipose tissue fibrosis and exacerbates metabolic dysfunction (Figure 2).

### 3.1. Atms Expansion During Obesity

Initially, exposure of macrophages to saturated fatty acids in adipose tissue triggers a mild inflammatory response that promotes adipocyte lipid synthesis and hypertrophy. Concurrently, obese adipose tissue secretes cytokines enhance macrophage recruitment, thereby amplifying local inflammation and supporting adipose expansion [11,42,48]. In advanced obesity, immune cells undergo metabolic reprogramming that shapes a distinct microenvironment marked by hypoxia, lactate accumulation, and elevated FFA. Ectopic lipid deposition culminates in CLS, which are enriched with LAMs. These macrophages modulate adipose tissue homeostasis through multiple mechanisms, such as clearance of apoptotic adipocytes, lipid metabolism, extracellular matrix remodeling, and support of angiogenesis and adipogenesis [30,49,50]. Under physiological conditions, adipose tissue expansion adaptively limits hypoxia, inflammation, and fibrosis [51]. However, overnutrition leads to lipid accumulation within adipocytes, triggering meta-inflammation via two major mechanisms: activation of pattern recognition receptors (PRRs) and chemokine secretion. FFA activates TLR4 and TLR2, inducing C-C motif ligand 2 (CCL2) production from adipocytes [49,50], while triggering TLR2 also upregulates cyclooxygenase-2 (COX-2) and iNOS in macrophages [52,53]. Beyond FFA, dead adipocytes represent another source of inflammation, promoting monocyte recruitment [54,55] and releasing danger-associated molecular patterns (DAMPs) that stimulate the NLRP3 inflammasome through NOD-like receptors on ATMs, thereby enhancing IL-1β and IL-18 release [56,57]. In summary, an initial mild inflammatory response is beneficial for healthy adipose expansion, whereas in obesity, chronic inflammation becomes detrimental, driving macrophage-mediated metabolic dysfunction, including insulin resistance, hyperlipidemia, and hepatic steatosis.

### 3.2. Atms, Insulin and Insulin Resistance

In the adipose tissue microenvironment, a bidirectional causal relationship exists between the metabolic state of macrophages and systemic insulin resistance. Metabolic reprogramming in ATMs is a core determinant of their inflammatory phenotype, and inflammation serves as a critical link bridging obesity and insulin resistance. In the context of obesity and T2D, despite the presence of systemic insulin resistance, impaired insulin signaling in macrophages leads to upregulation of glucose transporter 1 (GLUT1). This enhances glucose uptake and glycolysis, driving polarization toward a pro-inflammatory M1 phenotype. This metabolic shift fuels the pentose phosphate pathway (PPP) to generate abundant ROS and utilizes a broken tricarboxylic acid (TCA) cycle to accumulate succinate, thereby activating the NLRP3 inflammasome. The subsequent robust release of cytokines such as IL-1β and TNF-α exacerbates insulin resistance [58,59]. Conversely, while the traditional view posits that inflammation induces insulin resistance, the direction of causality is being reexamined. A study demonstrated that insulin resistance in adipose tissue precedes macrophage accumulation and the onset of inflammation [60]. Furthermore, under conditions of insulin resistance or obesity, the adipose tissue microenvironment is abundant with FFA. In response, ATMs employ ATP-binding cassette transporter G1 (ABCG1) to transfer pro-inflammatory saturated fatty acids from the phospholipid bilayer of the cell membrane into lipid droplets for storage. This sequestration neutralizes their toxicity, reduces membrane fluidity, and suppresses inflammatory responses. This research indicates that the state of insulin resistance imposes a requirement for metabolic adaptation on ATMs [61].

### 3.3. Failure of Adipose Tissue Remodeling

During adipose tissue expansion, impaired angiogenesis can induce hypoxia, inflammation, and the accumulation of senescent cells [62,63]. As adipocytes exceed a critical size, their capacity for adaptive hypertrophy and hyperplasia is compromised, leading to ectopic lipid leakage. This persistent expansion culminates in a state of “adipocyte remodeling failure,” which promotes ectopic lipid deposition and triggers local chronic inflammation along with systemic immune dysregulation, thereby increasing susceptibility to infections and mortality [64]. Lipid leakage and cell death attract monocytes and macrophages, amplifying tissue inflammation. Locally, NOD-like receptors sense DAMPs from damaged adipocytes and activate the NLRP3 inflammasome in ATMs, stimulating IL-1β and IL-18 secretion, a process shown to aggravate inflammation in ApoE^−/−^ mice in our previous studies [65]. Concurrently, FFAs and DAMPs further promote macrophage recruitment and the formation of CLS, which serve as histological markers of chronic inflammation and key sources of pro-inflammatory cytokines [21,66]. CLS consists not only of pro-inflammatory CD11c^+^ macrophages but also LAMs. These CLS-associated ATMs undergo metabolic adaptations that enhance lipid processing, showing upregulated expression of lysosomal genes and lipid metabolic genes [14,42,67]. Importantly, inflammation in this context originates from lipid-driven activation of intracellular inflammatory pathways in macrophages, rather than from prior inflammatory signaling upregulating lipid-handling genes [42]. The unfolded protein response (UPR), macrophage function, and inflammatory signaling are closely interconnected. At its center is the ER, an organelle essential for protein folding and metabolic regulation. Metabolic disturbances induce ER stress, activating the canonical UPR branches to restore homeostasis. IRE1α, a crucial transmembrane protein, activation represses adaptive thermogenesis by degrading peroxisome proliferator-activated receptor γ coactivator 1-α (PGC1α) mRNA, linking it to inflammatory pathways [2]. Further expanding its role, studies show that pharmacological inhibition or deletion of IRE1 enhances brown fat activity, promotes white fat browning, and increases energy expenditure [68]. Notably, Inositol-requiring enzyme 1α (IRE1α) deficiency also enhances M2 macrophage polarization in a cell-autonomous manner [69]. Mechanistically, this stress response may involve altered IRE1α cluster dynamics and interactions with stress granules, which can impair X-box binding protein-1 (XBP1) mRNA splicing and its downstream transcriptional programs [70].

### 3.4. Macrophage Under Metabolic Stress

MMes are induced by hyperglycemia, hyperinsulinemia, and high FFAs and perform lipid clearance (forming CLS, using lysosomal exocytosis marked by LAMP1/2) while responding to inflammatory signals [14]. Their dual role is regulated by adipocyte-derived factors: lipid-loaded extracellular vesicles enhance lipid-processing genes, and saturated fatty acids activate pro-inflammatory pathways via TLRs [42]. This balance dictates adipose homeostasis. Early in obesity, inflammation is prominent. With failing adipocyte hypertrophy, MMes co-upregulate lipid metabolic and inflammatory genes [10]. Eventually, excessive lipid overload compromises their functional balance, leading to unabated inflammation, M1 macrophage accumulation, and severe insulin resistance [11].

### 3.5. ATMs and WAT Browning

Beyond their well-established roles in inflammation and insulin resistance, ATMs have emerged as critical regulators of WAT browning, including multilocular lipid droplets and uncoupling protein 1 (UCP1)-mediated thermogenesis. This functional axis positions ATMs as direct modulators of whole-body energy expenditure, with significant implications for obesity pathogenesis and treatment. Current evidence suggests that inflammatory macrophages and M2 macrophages exert opposing effects on adaptive thermogenesis, promoting and inhibiting it, respectively. The underlying mechanisms involve chemokine-mediated recruitment, miRNAs, mitochondrial function, and direct suppression of UCP1 expression [71,72,73,74]. A landmark 2024 study revealed that ATM can themselves express UCP1 under specific conditions. Myeloid-specific deletion of HIF-1α led to the emergence of a UCP1^+^ ATM population in WAT that produced significant heat and directly mediated adipocyte lipolysis in co-culture experiments. This discovery fundamentally expands our understanding of macrophage-adipocyte metabolic crosstalk [75]. Multiple recent studies demonstrate that pharmacologically reprogramming ATM can rescue WAT browning in obesity. Rhein, a natural compound derived from rhubarb, targets macrophage SIRT2 to inhibit NLRP3 inflammasome activation, thereby promoting adipocyte thermogenesis; myeloid-specific knockdown of SIRT2 abolishes these metabolic benefits [76]. Sarcosine activated the GCN2 signaling pathway to enhance anti-inflammatory macrophage polarization, promoting adipose thermogenesis [77]. Collectively, these findings establish ATMs as regulators of WAT browning and anti-thermogenic under inflammatory conditions. 

### 3.6. Metabolic Gene Signatures ATMs in Obesity

Recent advances in spatial transcriptomics have fundamentally shifted our understanding of ATM heterogeneity by mapping metabolic gene expression onto anatomical localization. Prior to the spatial era, single-cell RNA-seq (scRNA-seq) had identified diverse ATM subsets in obesity, including TREM2^+^ lipid-associated macrophages (LAMs) and inflammatory macrophages (IMs), but lacked spatial context [78,79]. The landmark study by Stansbury et al. integrated scRNA-seq, spatial transcriptomics, and imaging in murine adipose tissue across diet-induced obesity progression. This multimodal approach revealed that LAMs disproportionately accumulate within CLS surrounding dead or stressed adipocytes. Critically, a transitional population termed “pre-LAMs”—transcriptionally intermediate between monocytes and mature LAMs—was found to be spatially associated with nascent CLS in early obesity. Spatial colocalization analysis identified enrichment of lipid metabolism-related ligand-receptor pairs (Apoe, Lrp1, Lpl, App) among monocytes, pre-LAMs, and LAMs within the CLS microenvironment, directly implicating lipid metabolic signaling in CLS formation and ATMs lineage progression [80]. A study by Weinstock et al. further refined this spatial-metabolic framework, distinguishing two functional LAMs subsets based on spatial localization: “adaptive LAMs”, enriched in CLSs and characterized by lysosomal lipid–catabolic genes (*Lipa*, *Lpl*, *Pparg*, *Scarb2*), and “maladaptive LAMs”, predominantly found as isolated or paired cells in interstitial spaces and marked by inflammatory genes (*Nlrp3*, *Tlr2*) [81]. This spatial segregation suggests that lysosomal lipid metabolism is a spatially restricted adaptive program that becomes maladaptive only when dysregulated or sustained. In human obesity, a very recent spatial transcriptomics study of 489 individuals by Zeng et al. identified LAMA4-ITGA9/ITGB1 signaling as a key mediator of adipocyte–macrophage crosstalk in CLS. LAMA4 expression correlated with BMI, macrophage activation, and ECM remodeling, and was spatially validated by multiplex immunofluorescence. This work provides direct human evidence that spatially confined ligand–receptor interactions couple adipocyte metabolic stress to ATMs inflammatory reprogramming [82]. Collectively, these spatial transcriptomic findings demonstrate that glycolysis- and PPP-driven pro-inflammatory ATMs are likely enriched in interstitial spaces during early obesity, whereas LAMs executing lysosomal lipid catabolism are spatially constrained to CLSs. This spatial–metabolic integration refines our understanding of ATM heterogeneity as potential targets for subset-selective therapeutic intervention.

## 4. Macrophage Metabolic Reprogramming

### 4.1. Key Metabolic Drivers of ATMs Inflammatory Polarization in Obesity: Causative vs. Adaptive Changes

Metabolic reprogramming governs macrophage activation. Under physiological conditions, this process is rhythmic and tightly regulated. In the context of obesity and overnutrition, however, macrophages adapt their metabolic phenotype and plasticity in response to environmental changes to help maintain systemic homeostasis. Among the numerous metabolic alterations observed in obese ATMs, three pathways emerge as causative drivers of pro-inflammatory activation. First, glycolytic flux, particularly the HIF-1α, is an early and essential factor that directly licenses IL-1β production. Second, the pentose PPP supplies NADPH for NOX2-dependent ROS generation, linking glucose metabolism to oxidative burst capacity. Third, while fatty acid oxidation (FAO) supports oxidative phosphorylation in resident anti-inflammatory ATMs, its overload—not the pathway itself—triggers mitochondrial stress and inflammasome activation. In contrast, several metabolic features are downstream consequences rather than initiating events. These include the suppression of oxidative phosphorylation (OXPHOS), accumulation of lipid droplets, and adoption of the LAMs transcriptional program. Lactate occupies an intermediate position: although primarily a byproduct of aerobic glycolysis, it can feedback through GPR81 to sustain HIF-1α stabilization. 

### 4.2. Glucose Metabolism 

**Figure 3:**
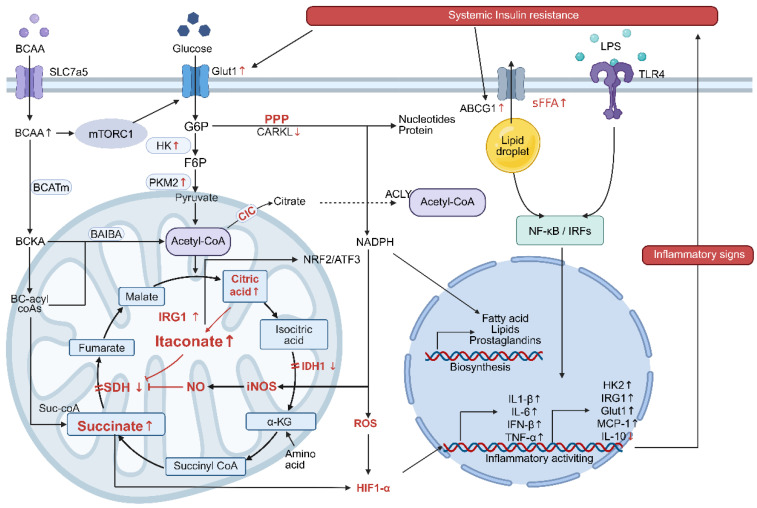
Metabolic reprogramming of ATMs in glucose metabolism. Under the inflammatory microenvironment, ATMs significantly enhance their glycolytic flux to meet energy demands. Inflammatory ATMs exhibit enhanced glycolytic flux for ATP generation while having an interrupted mitochondrial TCA cycle, driving the accumulation of succinate, itaconate, and citrate. Concurrent activation of the pentose phosphate pathway facilitates nicotinamide adenine dinucleotide phosphate (NADPH) production, supporting inflammatory biosynthetic demands and controlling the redox balance. In brief, inflammatory macrophages are more glycolytically active and markedly upregulate activities that are associated with inflammatory responses.

Glucose metabolism serves as a fundamental source of energy for cellular functions. Dietary glucose is converted into energy through three major pathways: glycolysis, PPP and the tricarboxylic acid (TCA) cycle. In resting macrophages, these pathways coordinate to produce adenosine 5’-triphosphate (ATP) and support basal metabolic activities [83] (Figure 3). 

### 4.3. Glycolysis and the PPP

Metabolic reprogramming in pro-inflammatory macrophages is characterized by the synergistic activation of glycolysis and the PPP [84]. Single-cell RNA sequencing analyses reveal that OXPHOS serves as a key discriminative feature among tissue-resident macrophages under steady-state conditions. Notably, deficiency in mitochondrial transcription factor A, which controls the replication and transcription of mitochondrial DNA, attenuates the pro-inflammatory activation of lipid-laden macrophages in WAT, conferring protection against insulin resistance and hepatic steatosis. Thus, OXPHOS not only distinguishes macrophage subsets across tissues but also supports lipid handling and pro-inflammatory functions in ATMs [85]. During pro-inflammatory polarization, enhanced glycolytic flux coincides with suppressed mitochondrial OXPHOS. This metabolic reprogramming swiftly produces ATP to support increased energy requirements, simultaneously diverting glycolytic intermediates into the PPP, thereby creating a unique metabolic environment that supports pro-inflammatory responses. A core mechanism linking metabolism to inflammation involves the oxidative PPP and its key enzyme, glucose-6-phosphate dehydrogenase (G6PD), which generates NADPH to support both ROS production and antioxidant defenses, thereby tuning inflammatory responses [86,87,88]. In obese adipose tissue, hypoxia-induced HIF-1α amplifies this circuit by upregulating glycolytic enzymes and G6PD, increasing PPP activity [89]. Mechanistically, this reprogramming activates the NLRP3 inflammasome through several convergent paths: direct binding and activation by glycolytic hexokinase 1 (HK1), promotion of assembly by PPP-derived ROS, and mtROS-driven caspase-1 activation due to Nrf2-impaired mitophagy and subsequent damaged mitochondrial accumulation, leading to IL-1β maturation [90,91,92]. The traditional metabolic paradigm, which categorizes glycolysis as pro-inflammatory and FAO as anti-inflammatory, is likely an oversimplification, leading to a growing number of conflicting observations. Generally, glycolysis supports pro-inflammatory functions in macrophages of hypertrophic adipose tissues, with the underlying mechanism involving HIF-1α and the induction of inflammatory cytokine expression. However, the functional outcome of macrophages was also determined by the utilization of downstream metabolic pathways [90,93]. The differential regulation of PPP may be the basis of their functional heterogeneity. Oxidative PPP activity was enhanced in M1 macrophages, and NADPH production supported ROS production and inflammasome activation. M2 macrophages inhibit oxidative PPP activity through STAT6-dependent G6PD phosphorylation, thereby limiting NADPH production. In addition, M1 showed a broken TCA cycle, leading to the accumulation of succinic acid and citric acid, stabilizing HIF-1α and driving the expression of inflammatory genes, such as IL-1β; m2 maintained a complete TCA cycle coupled with OXPHOS. This is precisely the difference in the utilization of these downstream metabolic pathways (PPP and TCA), which determine the functional heterogeneity of ATMs more than the rate of glycolysis.

### 4.4. Disruption of the TCA Cycle

Anti-inflammatory macrophages maintain an intact TCA cycle coupled to OXPHOS. In contrast, pro-inflammatory macrophages display a fragmented TCA cycle, with breaks at two key enzymatic nodes. The first disruption occurs at isocitrate dehydrogenase 1 (IDH1), impairing the conversion of isocitrate to α-KG and leading to citrate accumulation. Cytosolic citrate is cleaved by ATP-citrate lyase (ACLY) to generate acetyl-CoA, which fuels the synthesis of pro-inflammatory lipids, such as prostaglandins, and facilitates histone acetylation to promote inflammatory gene expression [94]. Additionally, acetylation of the mitochondrial citrate carrier (CIC) enhances its activity, promoting citrate efflux and establishing a positive feedback loop that amplifies inflammatory signaling [95,96].

A second break occurs at succinate dehydrogenase (SDH), resulting in succinate accumulation. Succinate inhibits prolyl hydroxylases (PHDs), thereby stabilizing HIF-1α and enhancing the expression of IL-1β and other inflammatory genes. Furthermore, succinate drives mitochondrial ROS production via reverse electron transport (RET), synergizing with NLRP3 inflammasome activation [97,98,99]. Glycolytic activation coupled with TCA cycle disruption augments pro-inflammatory cytokine production through HIF-1α, while impaired OXPHOS promotes the accumulation of lipid intermediates that activate TLR4/NF-κB signaling [83,100,101]. In obesity, FFA released by adipocytes are internalized via CD36 in ATMs, inducing mitochondrial uncoupling and ER stress, which correlate with NLRP3 activation [14,57]. 

Branched-chain amino acid (BCAA) metabolism further reinforces this inflammatory program: leucine activates mTORC1 to enhance ACLY phosphorylation and Glut1 expression, boosting glycolysis and acetyl-CoA production. Meanwhile, isoleucine-derived malonyl-CoA inhibits carnitine palmitoyltransferase 1 (CPT1), further promoting lipid synthesis and inflammatory activation [102,103,104]. 

In addition, metabolic reprogramming following SDH inhibition promotes compensatory activation of the itaconic acid biosynthesis pathway [105]. Itaconic acid inhibits mitochondrial complex II by targeting SDH, thereby reducing the basal oxygen consumption rate (OCR) and shifting macrophage metabolism toward glycolysis. Concurrently, itaconate alkylates cysteine residues on Keap1, stabilizing the transcription factor Nrf2 and promoting the expression of antioxidant genes, including hemeoxygenase 1 (HO-1) and NAD(P)H Quinone Dehydrogenase 1 (NQO1), which attenuates ROS levels and alleviates oxidative stress [106]. Chen et al. demonstrated that Keap1-Nrf2 pathway activation modulates lipid metabolism in macrophages, diminishes oxidative stress, and restrains inflammatory activation, thereby ameliorating the progression of nonalcoholic fatty liver disease (NAFLD) [107]. Furthermore, macrophage-derived itaconate reduces lipid accumulation in hepatocytes and enhances OXPHOS by promoting FAO [108]. These findings indicate that itaconic acid exerts pleiotropic roles in antibacterial defense, anti-inflammatory regulation, and lipid metabolism. 

### 4.5. Lipid Metabolism

**Figure 4:**
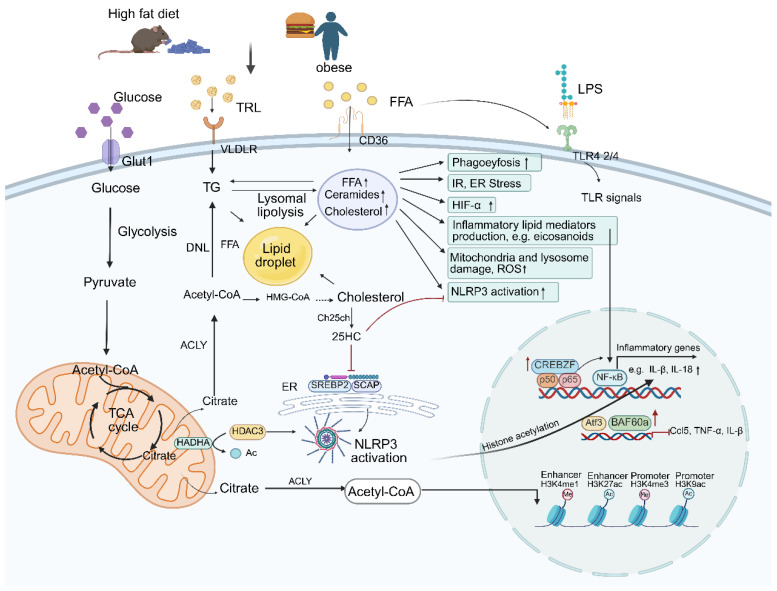
Metabolic reprogramming of ATMs in lipid metabolism. It highlights glucose-derived citrate partitioning into lipogenesis or histone acetylation, mitochondrial histone deacetylase 3 (HDAC3) activity, enhancing NLRP3-driven IL-1β release, and the counter-regulatory role of 25-Hydroxycholesterol (25-HC). It further integrates how lipid overload activates TLR/NF-κB signaling and how transcription factors (CREBZF, Atf3/BAF60a) modulate inflammatory gene expression. TRL, triglyceride-rich lipoproteins. Glut1, glucose transporter 1. IR, insulin resistance. 25HC, 25-hydroxycholesterol. DNL, de novo lipogenesis.

Macrophage lipid metabolism is highly plastic and adapts to physiological and pathological stimuli, thereby shaping macrophage activation and function. Dysregulated lipid metabolism contributes to macrophage dysfunction, exemplified by cholesterol accumulation in arterial macrophages, promoting atherosclerosis, or pathogen-induced alterations in host cholesterol metabolism, facilitating viral survival [109] (Figure 4). When triglyceride (TG) levels are high, triglyceride-rich lipoproteins (TRLs) can be taken up by macrophages via VLDLR and converted into FFAs. Excess FFAs, together with intracellular cholesterol, synergistically activate DGAT1/2 (diacylglycerol acyltransferase), re-synthesize TG and form lipid droplets, thereby promoting the transformation of macrophages into foam cells. Excess FFAs activate NF-κB and NLRP3 inflammasome, and promote the expression of pro-inflammatory factors such as IL-1β, IL-6, and TNF-α [110]. Omega-3 fatty acids include docosahexaenoic acid (DHA) and eicosapentaenoic acid (EPA). DHA can promote the degradation of triglycerides in lipid droplets within macrophages, thereby inhibiting macrophage foam cell formation, while EPA can antagonize the effect of DHA [111]. Omega-6 fatty acids include linoleic acid (LA), γ-linolenic acid (GLA), arachidonic acid (AA), etc. Omega-6 (especially AA) generates strong pro-inflammatory lipid mediators such as prostaglandin E2 (PGE2) under the catalysis of cyclooxygenase (COX-2) and lipoxygenase (LOX). These mediators can directly bind to corresponding receptors on the surface of macrophages, activate the NF-κB signaling pathway, and promote the expression of inflammatory factors. Omega-6 can also be catalyzed by lipoxygenase to generate lipoxin A4 (LXA4), which can inhibit the activation of the NF-κB in macrophages, up-regulate the expression of anti-inflammatory factors, and promote tissue repair [112]. Recent studies have demonstrated that genetic or dietary interventions aimed at reducing membrane ω-6 polyunsaturated fatty acid levels lead to impaired TG in WAT, accompanied by ectopic lipid deposition and insulin resistance [113]. The balance of Omega-6/Omega-3 ratio in the diet is crucial for maintaining macrophage immune homeostasis; an excessively high ratio will significantly amplify the pro-inflammatory effect of Omega-6 and exacerbate macrophage-mediated inflammatory responses [114]. Kasparas et al. showed that ATMs from obese mice and humans exhibit upregulated de novo phosphatidylcholine biosynthesis. Macrophage-specific ablation of CCTα, the rate-limiting enzyme in this pathway, protects against obesity-induced WAT inflammation and insulin resistance, which is related to reduced ER stress and inflammation in response to palmitate [115]. Under starvation or ketogenic diet conditions, high concentrations of β-hydroxybutyrate (β-HB) can inhibit the NLRP3 inflammasome and directly suppress macrophage inflammation [116]. In the state of diabetic nephropathy, the ketone body metabolite acetoacetate (AcAc) increases and activates the MIF/ERK-signaling pathway in macrophages, driving the polarization of macrophages towards the pro-inflammatory M1 type and exerting a pro-inflammatory effect [117].

### 4.6. Lipid Biosynthesis Is Essential for Membrane Remodeling and the Synthesis of Inflammatory Mediators

In pro-inflammatory macrophages, lipids serve as precursors for inflammatory mediators and enhance inflammasome activation. A key regulator of lipogenesis is fatty acid synthase (FAS), which is essential for membrane remodeling. FAS deficiency alters plasma membrane composition and impedes Rho guanosine triphosphatases (Rho GTPase) trafficking, thereby attenuating inflammatory signaling [118]. For instance, TLR4 activation by LPS enhances *de novo* lipogenesis (DNL) in macrophages. DNL provides a critical source of endogenous fatty acids that supports phagocytosis upon microbial challenge [109]. Genetically or pharmacologically depleting FASN attenuates NLRP3 inflammasome signaling in BMDMs by reducing the palmitoylation of its target proteins through toll-like receptor [119]. Exogenous saturated fatty acids further contribute to inflammasome activation by elevating saturated phosphatidylcholine levels [120]. Our studies show that ApoE^−/−^ mice are resistant to obesity and exhibit enhanced systemic glucose tolerance and insulin sensitivity. However, their adipose tissue displays signs of metabolic inflammation, including pro-inflammatory macrophage activation and increased levels of TNFα and IL-1β [65].

### 4.7. FAO Promotes Oxidative Metabolism and Histone Acetylation

FAO is a central bioenergetic and epigenetic pathway in anti-inflammatory macrophages. M2 polarization relies on mitochondrial oxidative metabolism, wherein FAO generates ATP, acetyl-CoA, and NAD^+^ to support alternative activation [121]. FAO-driven oxidative metabolism supports M2 activation through multiple mechanisms: M2 macrophages exhibit heightened FAO dependency, and increased oxidative metabolism elevates acetyl-CoA levels, enhancing histone acetylation and facilitating IL-4 signaling [122,123]. The Akt-mTORC1 pathway regulates ACLY, a key enzyme in acetyl-CoA synthesis, further promoting histone acetylation and anti-inflammatory gene expression. Etomoxir, an inhibitor of CPT1a, significantly reduces expression of M2 markers but minimally affects M1 markers, suggesting that FAO inhibition directly impairs metabolic reprogramming in macrophages [124].

However, the requirement of FAO for M2 polarization remains controversial. Myeloid-specific Cpt2 knockout BMDMs polarize normally into M2 macrophages upon IL-4 stimulation, and etomoxir suppresses M2 marker expression in both control and Cpt2-deficient cells, indicating that FAO is dispensable for M2 polarization [125].

Conversely, Nox4-mediated FAO facilitates NLRP3 inflammasome activation. Nox4 deficiency reduces CPT1 expression and suppresses both FAO and NLRP3 activation. Notably, in obesity, adipocyte-derived FFAs accumulate in ATMs via CD36-mediated uptake, inducing mitochondrial uncoupling and ER stress-events closely linked to NLRP3 activation [14,57]. Myeloid-specific CD36 deletion does not reduce lipid accumulation or inflammation in HFD-fed mice, indicating that targeting CD36 alone is insufficient to prevent lipid overload in ATMs [126]. Moreover, CD36 mediates oxLDL-induced mitochondrial ROS production in macrophages, which impairs mitochondrial function, enhances superoxide generation, and activates NF-κB to drive inflammation [127]. These findings suggest that dysregulated fatty acid metabolism and oxidative stress collaboratively shape macrophage inflammatory responses. In summary, although FAO inhibition does not completely abrogate M2 polarization, these studies highlight that metabolic reprogramming should not be viewed as a sole determinant of macrophage function.

### 4.8. Cholesterol

Macrophages can transfer free cholesterol to HDL through mechanisms such as ATP-binding cassette G1 (ABCG1), which is then transferred to the liver, a process known as reverse cholesterol transport (RCT). This inhibits the transformation of macrophages into foam cells and delays the formation of atherosclerotic plaques [128]. When the content of free cholesterol in HDL particles is too high, free cholesterol is transferred to macrophages through bidirectional transport by SR-BI, leading to lipid accumulation in macrophages, promoting foam cell formation and AS plaque formation [129]. Lipid fluxes in adipose tissue drive obesity-associated inflammation, yet the underlying mechanisms remain incompletely understood. Myeloid-specific deletion of Fasn in mice prevents diet-induced insulin resistance, reduces macrophage recruitment to adipose tissue, and suppresses chronic inflammation. These protective effects are attributed to FAS deficiency-induced changes in membrane order and composition, impaired plasma membrane cholesterol retention, and disrupted Rho GTPase trafficking, highlighting cholesterol as an essential mediator of inflammatory signaling [130]. Dahik et al. demonstrate that ABCG1 on ATMs governs their inflammatory response to circulating saturated fatty acids, which are abundant in obesity. Myeloid-specific Abcg1 knockout in obese mice attenuated markers of inflammation, altered fatty acid metabolism, and reduced the accumulation of LAMs-findings that were mirrored in human samples [61].

Recruited monocytes differentiate into macrophages that proliferate and adopt a pro-inflammatory state. These macrophages release inflammatory mediators that activate the vascular endothelium, further promoting monocyte recruitment and perpetuating chronic arterial inflammation. Foam cell formation occurs via phagocytosis of oxidized low-density lipoprotein (ox-LDL). Accumulation of foam cells expands the plaque lipid core, increases mechanical instability, and promotes cholesterol crystal deposition, which activates the NLRP3 inflammasome and exacerbates local inflammation—an early event in atherosclerotic lesion development [131,132]. In contrast, anti-inflammatory macrophages promote cholesterol efflux, clear apoptotic cells, secrete IL-10, and help maintain plaque stability [133]. As key innate immune effector cells, macrophages require rapid membrane expansion for pathogen phagocytosis. TLR4 signaling is critical for lipopolysaccharides (LPS)-induced phagocytic responses and stimulates mechanistic target of rapamycin complex 1 (mTORC1) activity, which in turn promotes proteolytic maturation of sterol regulatory element-binding protein (SREBP) transcription factors [134]. Through SREBP-1a, mTORC1 upregulates lipogenic genes supporting fatty acid and cholesterol synthesis, thereby maintaining the lipid homeostasis essential for phagocytosis. Loss of SREBP-1a disrupts lipid raft architecture and impairs actin cytoskeleton interactions, significantly compromising phagocytic efficiency. Recent studies have shown that accumulated 25-HC, an oxidation product of cholesterol generated by 25-hydroxylase (CH25H), accumulates in the lysosomes of macrophages and competes with cholesterol to bind to the lysosomal-localized signal protein GPR155 to inhibit mTORC1 activation and enhancing the activation of AMPKα to enhance mitochondrial function and promote OXPHOS [135]. Notably, however, 25-HC production in activated macrophages has also been shown to amplify inflammatory phenotypes and promote atherogenesis [136].

## 5. Lactic Acid and Macrophage Metabolism

**Figure 5:**
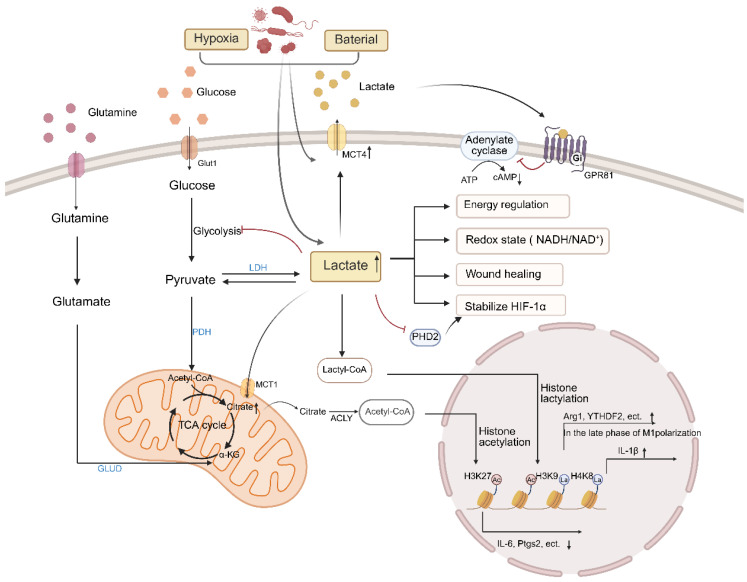
Lactate in ATMs metabolic reprogramming. Under obese conditions, both hypoxic and bacterial stimuli enhance the activity of lactate transporters monocarboxylate transporter 1 and 4 (MCT1/4), increasing lactate uptake. Derived primarily from glycolysis and glutaminolysis, lactate can be converted to lactoyl-CoA, which drives histone lactylation to promote the expression of the pro-inflammatory cytokine IL-1β. During later M1 polarization, lactylation facilitates an M2-like gene expression profile (e.g., *Arg1*, *Retnla*). Conversely, lactate can also suppress inflammation by promoting histone H3K27 acetylation, representing an alternative immunosuppressive phenotype. Additionally, lactate-derived citrate fuels the TCA cycle and helps maintain redox balance. Lactate further stabilizes HIF-1α by binding to and inhibiting the catalytic domain of prolyl hydroxylase domain 2 (PHD2). Glut1, glucose transporter 1. MCT, monocarboxylate transporter. PDH, pyruvate dehydrogenase. GLUD, glutamate dehydrogenase. ACLY, ATP-citrate lyase. PHD2, proline hydroxylase domain 2.

Lactic acid was initially thought to be a metabolic waste product. Excessive accumulation of lactic acid caused by tissue hypoxia or mitochondrial dysfunction may lead to lactic acidosis, and metabolic disorders. Lactic acid generated by hypoxic adipose tissue not only regulates macrophage phenotypic switching through monocarboxylate transporters (MCTs) but also activates the mTORC1 signaling axis via GPR81 receptors, synergistically promoting pro-inflammatory cytokines [137] (Figure 5). 

The synergistic activity of MCT1-4 promotes the shuttle of lactic acid between glycolytic cells and oxidative cells, which is a key factor in the homeostasis of lactic acid in different tissues [138]. Lactate receptor GPR81 regulates the transport of lactate in the plasma membrane and cells, thereby inhibiting cAMP and PKA-mediated signal transduction, mainly inhibiting lipolysis in adipocytes [139]. It was found that lactic acid, as the key fuel of TCA cycle as a metabolic raw material for macrophages, together with pyruvate, acts as a cyclic redox buffer to balance the NADH/NAD ratio between cells and tissues and maintain the balance of cell redox status in whole body tissues [140]. Lactic acid promotes the expression of anti-inflammatory related genes (such as Arg1) in the late stage of pro-inflammatory macrophage polarization by inducing histone lysine lactate [141]. In addition, lactic acid also promotes the induction and activation of epigenetic reprogramming such as the immunosuppressive gene NR4A member 1 (NR4A1) by promoting the acetylation of histone H3K27, leading to the transcriptional inhibition of pro-inflammatory responses in macrophages, and the influx or accumulation of lactic acid is necessary to mediate the anti-inflammatory effects of lactic acid [142]. 

Feng et al. identified adipocytes as the primary source of lactate in WAT, a process that appears to be largely independent of glucose metabolism. Their studies showed that adipocyte-derived lactate directly targets PHD2 in macrophage, leading to HIF-1α stabilization and activation, which in turn drives IL-1β expression and exacerbates local inflammation. Notably, lactate levels within WAT were positively correlated with markers of insulin resistance [143,144]. In contrast, other studies have reported that moderate supplementation with L-lactate suppresses M1 polarization of adipose tissue macrophages through activation of the GPR132–PKA–AMPKα cascade, thereby ameliorating obesity-related insulin resistance [145]. These divergent findings likely stem from differences in experimental design that yield disparate local lactate concentrations. While a unifying explanation remains elusive, these observations collectively point to a potential avenue for therapeutic intervention in obesity and insulin resistance. 

## 6. Mechanisms of Macrophages Metabolic Reprogramming 

### 6.1. Regulatory Factors of Environmental Sensing and Metabolic Response

#### 6.1.1. Krüppel-Like Factor (KLF)

Localized mainly in the nucleus, KLF is a key regulator of a variety of cellular processes, involved in the maintenance of systemic and tissue homeostasis, and plays a central role in metabolic regulation and modulation of immune cell function. In macrophages, KLF shape phenotypic and functional diversity by controlling cell differentiation, polarization, and metabolic reprogramming [146,147].

Kruppel-like factor 2/4 (KLF2/4) can control the expression of genes required for the identity of tissue macrophages, especially. Regarding Kruppel-like factor 2 (KLF2), which is highly expressed in the large cavity macrophage (LCM), it regulates the key transcription factors GATA-binding protein 6 (GATA6) and retinotic acid receptor (RAR) required for the differentiation of the LCM, thereby maintaining the LCM identity and function [148,149]. Kruppel-like factor 4 (KLF4) can regulate macrophage polarization and metabolic phenotype. KLF4 knockdown induces inflammatory factor expression in different macrophage subtypes. It exerts anti-fibrotic effects through IL-10 in alternative activation macrophages [150]. Moreover, KLF4 knockdown promotes intestinal myofibroblast cells IMF proliferation. In addition, KLF4 is essential for the development of alveolar macrophage, and its deficiency results in impaired differentiation and reduced numbers of AM [148].

#### 6.1.2. Interferon Regulatory Factors

Interferon regulatory factors (IRFs) encompass a family of transcription factors critical for multiple immune functions, such as lymphopoiesis, macrophage differentiation, and innate immunity regulation, acting as pivotal effectors in TLR signaling pathways [151,152,153].

IRF3 functions synergistically across multiple cellular compartments to prevent the development of obesity-related metabolic abnormalities [154]. For adipocytes, IRF3 limits adipogenesis by controlling peroxisome proliferator-activated receptor γ (PPARγ), which is required to maintain WAT and BAT function [155]. 

In macrophages, IRF3 inhibits inflammatory activation through the IFNβ/IL-10 axis, thereby suppressing adipose tissue inflammation during WAT expansion. Research has demonstrated that a SENP-mediated reduction in IRF4 SUMOylation promotes macrophage M2 polarization, enhancing esophageal squamous cell carcinoma (ESCC) cell migration, invasion, and in vivo progression, thereby identifying a new target for ESCC immunotherapy [156]. IRF4 plays a significant protective role in the development of neointimal hyperplasia by regulating macrophage polarization, suggesting that artesunate could be developed as a novel therapeutic strategy for vascular restenosis [157]. Beyond these findings, research also shows that TANK-binding kinase 1 (TBK1), an important kinase involved in innate immunity and tumor development, partially regulates macrophage polarization in response to macrophage-colony-stimulating factor (M-CSF) stimulation through the IRF5/IRF4 axis [158]. 

IRF5 is a critical hub factor connecting metabolic reprogramming and inflammatory polarization of ATMs. In the obese energy-excess microenvironment, IRF5 drives ATMs dysfunction via a dual mechanism: on the one hand, as a classical pro-inflammatory transcription factor, it promotes ATMs polarization toward the M1 phenotype and upregulates the expression of inflammatory cytokines [159]; on the other hand, it transcriptionally represses the expression of the mitochondrial structural protein GHITM through a non-canonical pathway, disrupts the mitochondrial cristae structure, and impairs the oxidative phosphorylation capacity of ATMs [160]. This IRF5-mediated pro-inflammation and metabolic suppression state shifts ATMs from a protective, highly oxidative phenotype to a metabolically detrimental pro-inflammatory phenotype, thereby exacerbating adipose tissue inflammation and insulin resistance [161]. 

#### 6.1.3. Hypoxia-Inducible Factor (HIF) in Macrophages

Hypoxia occurs within expanded adipose tissue in obese individuals and in animal models of obesity. Much of this hypoxia is due to increased adipocyte size, decreased adipose tissue vascularization, and increased fatty acid metabolism that consumes oxygen. In addition, in the early stages of obesity resulting from an HFD, adipocytes respire uncoupling, resulting in increased oxygen consumption and adipocyte hypoxia. Decreasing oxygen levels induce immune cells, including macrophage metabolic reprogramming to sustain ATP production. 

HIF is a critical transcriptional regulator of immunity and inflammation and regulates macrophage reprogramming by targeting metabolism-associated enzymes, as well as promoting inflammatory responses [83]. HIF-1α is involved in the induction of glycolytic enzymes, which promote the selection of glycolytic pathways used for ATP production. HIF-1α promotes the expression of lactate dehydrogenase and pyruvate dehydrogenase kinase to support glycolytic pathways and limit OXPHOS [162]; as hypoxia is a well-known driver of glycolysis, an oxygen deficit results in limited OXPHOS. It also enhances the expression of hexokinase 2, glucose-6-phosphate isomerase, and pyruvate kinase M2, which promotes the conversion of glycolysis and promotes the activation of the NLRP3 inflammasome [163]. In the context of metabolic reprogramming, the activation of HIF-1 signaling further drives the expression of pro-inflammatory genes, including IL1B and iNOS, thereby contributing to the inflammatory phenotype commonly observed in metabolic diseases like diabetes. Furthermore, the glycolytic/HIF-1α axis is a key determinant of the inflammatory actions in IL-4-primed macrophages [164]. In vivo, myeloid-specific deletion of HIF-1α ameliorated HFD-induced insulin resistance following 18 weeks of feeding, without significantly affecting adipose tissue inflammation in the early phases [165,166].

Insulin resistance due to obesity can be alleviated by interfering with the activation of NLRP3 inflammatory vesicles, and more importantly, HIF-2α plays an important role in this process [167]. HIF-2α exerts more anti-inflammatory activity that inhibits macrophage activation by inhibiting pro-inflammatory mediators, such as mitochondrial ROS, which stabilize HIF-2α [168]. Accumulating evidence also indicates that HIF-2α signaling exerts pleiotropic regulatory functions in metabolically triggered inflammatory diseases, including cardiovascular disease, NASH, and obesity [167,169,170,171].

#### 6.1.4. CREBZF (CREB/ATF Bzip Transcription Factor)

CREBZF deficiency in macrophages attenuates macrophage infiltration in fat, pro-inflammatory activation, and hyperglycemia in obese mice. Co-culture assays have shown that macrophage CREBZF deficiency improves insulin sensitivity in primary adipocytes and adipose tissue [172].In addition, recent studies have revealed that CREBZF is also regulated by glucose signaling in brown fat through reversible acetylation mediated by CREB-binding protein (CBP)/p300 and HDAC3. Deficiency of CREBZF enhances glucose-induced thermogenesis and energy expenditure. CREBZF-mediated inhibition of thermogenesis represents a mechanism by which glucose regulates energy homeostasis, which may be required for the inhibition of browning and hyperactivation of thermogenesis [173].

#### 6.1.5. Nrf2

Nrf2 is a key antioxidant transcription factor. It plays a central role in maintaining cellular redox homeostasis and lipid metabolic homeostasis. In macrophages, Nrf2 is not only a key transcriptional activator of the antioxidant response, but also participates in the dynamic balance between inflammatory and antiviral responses by regulating metabolic reprogramming and innate immune pathways [92]. Nrf2 exerts broad anti-inflammatory effects by suppressing pro-inflammatory cytokines and their effectors [174,175]. Its activation is critical for the inhibition of IFN-β signaling by electrophilic Keap1 modifiers. Following macrophage activation, Nrf2 expression is elevated through a self-protective mechanism against oxidative stress-induced senescence, which is orchestrated by the kinase Mst1/2. Mst1/2 maintains Nrf2 protein stability by phosphorylating Keap1, thereby inhibiting Keap1 polymerization and its capacity to target Nrf2 for degradation. Loss of Mst1/2 significantly impairs macrophage resistance to oxidative stress, promoting premature cellular senescence and apoptosis that can be reversed by Nrf2 overexpression. Thus, the Mst1/2-Keap1-Nrf2 axis is essential for preserving redox homeostasis and preventing premature senescence in macrophages [92,176].

### 6.2. Post-Translational Modifications

#### 6.2.1. Histone Acetylation, Deacetylation, and Lactylation

Histone acetylation and deacetylation are pivotal epigenetic mechanisms that regulate chromatin architecture and gene transcription, thereby influencing metabolism and inflammation. In macrophages stimulated with LPS, citrate synthesis is upregulated and converted to acetyl-CoA by ACLY to modulate the transcription of inflammatory genes [177]. Concurrently, lactate serves as a key fuel for the TCA cycle, driving epigenetic reprogramming via histone H3K27 acetylation. This lactate-induced acetylation enables the expression of immunosuppressive genes, such as Nr4a1, which in turn suppresses pro-inflammatory responses and promotes long-term immunosuppression [142]. HFD significantly reduced the levels of acetyl CoA and/or the acetyl CoA: CoA ratio in WAT and had an impact on histone acetylation levels, which is consistent with the effect of ATP citrate lyase [178]. Separate research indicates that lipotoxicity upregulates HDAC3 expression and impairs mitochondrial oxidative capacity. This lipotoxicity-induced dysfunction can be prevented by a class I HDAC inhibitor, which ameliorates insulin resistance and curbs inflammatory cytokine secretion in muscle tissue [179].

Furthermore, recent studies collectively demonstrate that histone acetylation interfaces with metabolic reprogramming during macrophage-mediated inflammatory responses to promote inflammation resolution [180,181,182]. During inflammasome-driven inflammation, HDAC3 translocates to the mitochondria, where it deacetylates and suppresses the activity of HADHA, a critical enzyme involved in mitochondrial FAO. This modification facilitates macrophage adaptation to FAO and mitochondrial remodeling, ultimately enhancing IL-1β production [183]. Activation of HDACs leads to inflammation and fibrosis, which can be inhibited by pan-inhibitors of HDACs. HDAC3 was found to be a key regulator in inflammation and fibrosis in chronic kidney disease (CKD). Mechanistically, HDAC3 was found to modulate the acetylation of the Lys122 site of NF-kB p65 in macrophages and affect the onset and development of inflammation and fibrosis in CKD. Translationally, enzyme-activated inhibitors of HDAC3 were found to ameliorate UUO and I/R-induced renal fibrosis. Targeted inhibition of HDAC3 may be a new strategy for the treatment of CKD and also provides a new perspective on the treatment of CKD [184].

Emerging evidence indicates that inhibiting specific HDAC isoforms improves adipose tissue function and counteracts obesity [185,186,187,188]. Currently, five HDAC inhibitors have been approved for marketing, but all of them are used for cancer treatment in the clinic. Among the various subtypes of HDACs, HDAC3 has been found to exert both deacetylase activity and non-enzymatic function to mediate inflammatory signaling in macrophages. Protein hydrolysis-targeted chimera (PROTAC) technology was used to target the deacetylase-independent function of HDAC3. Investigators developed a potent and selective HDAC3-targeted PROTAC P7, which induced near-complete HDAC3 degradation at low micromolar concentrations in THP-1 cells and human primary macrophages. PROTAC P7 increased anti-inflammatory cytokine secretion in THP-1-derived M1-like macrophages. Importantly, PROTAC P7 reduced pro-inflammatory cytokine secretion in M1-like macrophages derived from human primary macrophages. This can be explained by the observed inhibition of macrophage classical activation. HDAC3-directed PROTAC P7 was shown to have anti-inflammatory activity and block macrophage polarization, demonstrating that this molecular mechanism can be targeted for small molecule therapy [189].

We identified the chromatin remodeling subunit BAF60a as a critical epigenetic checkpoint linking obesity-associated stress signals with metabolic inflammation and systemic energy homeostasis. BAF60a modulates the transcription of inflammatory mediators through the regulation of histone methylation and acetylation. Furthermore, we discovered that Atf3 functions as a downstream effector in BAF60a-mediated chromatin remodeling, playing a pivotal role in the transcriptional reprogramming of macrophage polarization and activation within adipose tissue [190]. In 2019, Zhao et al. demonstrated for the first time the existence of histone lysine lactylation (Kla) and showed that histone Kla is an in vivo PTM of proteins derived from lactate [141]. Lysine lactylation is highly expressed in promoter regions and promotes the binding of associated transcription factors to gene promoters to upregulate gene transcription. Histone lactonization and acetylation have different temporal dynamics. Histone lactylation is not required for the induction or repression of pro-inflammatory genes, but rather serves as a mechanism to initiate homeostatic gene expression in association with M2 macrophages [141]. Histone Kla is significantly elevated at the promoters of M2 genes during the late stages of polarization of stimulated M1 macrophages, suggesting that histone Kla may act as a lactate clock to promote the transition of macrophages from an inflammatory phenotype to a homeostatic phenotype. p300 may catalyze the Kla response, with a strong dependence on the p53 pathway. Several studies have demonstrated that p300/CBP regulates histone lactylation in macrophages and induced pluripotent stem cells (iPSCs) [191,192]. Among the components of these mechanisms, lactate coenzyme a is closely associated with enzymatic lactation, whereas lactate glutathione (LGSH) is involved in non-enzymatic lactation [138]. Zhang and other researchers found that histone Kla promotes the expression of M2 genes in M1 macrophages in the late stages of polarization after repair of collateral damage in response to inflammation through the potential histone Kla writing protein p300 [139].

#### 6.2.2. Protein Oxygen-Linked Glycosylation (O-Glcnac) Modification and Lipid Metabolism

O-GlcNAc transferase (OGT) catalyzes the O-linked N-acetylglucosamine (O-GlcNAc) modification of intracellular proteins, a PTM essential for metabolic homeostasis. Adipose tissue-specific OGT deletion exacerbates adipose tissue inflammation and impairs lipid storage in primary adipocytes. Pharmacological or genetic inhibition of OGT elevates FFA release from adipocytes, and conditioned medium from OGT-deficient adipocytes induces inflammatory gene expression in macrophages, suggesting that OGT loss promotes adipose inflammation partly via FFA-mediated intercellular communication. These findings indicate that O-GlcNAcylation supports healthy adipose expansion and restrains inflammation [193]. Under overnutrition, O-GlcNAc signaling is enhanced and acts to suppress macrophage pro-inflammatory activation. Myeloid-specific OGT deletion enhances pro-inflammatory polarization, adipose tissue inflammation, and lipolysis, leading to ectopic lipid deposition and systemic insulin resistance in HFD-fed mice. Mechanistically, OGT catalyzes O-GlcNAcylation of S6K1, which attenuates its phosphorylation and downstream mTORC1 signaling, thereby constraining macrophage inflammatory responses. Together, these results highlight a critical role for O-GlcNAc signaling in maintaining metabolic homeostasis during overnutrition [194]. Recent studies have also found that increased O-GlcNAc glycosylation of growth response 2 (EGR2) in limits CD8^+^ T cell-mediated anti-tumor responses and promotes tumor progression [195]. 

#### 6.2.3. Neddylation: A Dual Role in the Macrophage Inflammatory Response

Neddylation is a class of ubiquitination modifications that covalently attach NEDD8 to lysine residues in specific substrate proteins through a cascade of NAE, Ubc12, and Nedd8-E3 ligases, with an important target being the cullin subunit of the CRL (Cullin-RING E3 ligase). Neddylation inactivation of neddylation indirectly leads to the accumulation of CRL substrates, resulting in DNA damage and apoptosis. Neddylation inactivation would then inhibit NF-κB-mediated pro-inflammatory cytokine production in macrophages, and thus, the ubiquitination-like pathway can indirectly regulate and prevent sepsis associated with pro-inflammatory cytokine secretion [196,197]. Notably, hepatic and adipose tissues from obese mice exhibit elevated Neddylation activity, and pharmacological inhibition of Neddylation produces anti-obesity and glucose-lowering effects. For neddylation, while direct evidence of TLR4 in ATMs is limited, a study established a drug transport mechanism utilizing nanospheres coated with MLN4924 in RAW264.6. MLN49224, a compound that inhibits neddylation, enhances the healing of diabetic wounds by inhibiting the polarization of M1 macrophages and reducing the secretion of inflammatory factors [198]. 

Supporting a broad role in inflammation, genetic ablation of the Neddylation E2 enzyme (UBE2M) in macrophages markedly reduces the production of pro-inflammatory cytokines, including IL-1β, IL-6, and TNF-α. Recent studies have reported that UBE2M is essential for macrophage-induced obesity-associated inflammation. In mice lacking UBE2M macrophages, HFD-induced obesity, insulin resistance, and hepatic steatosis were greatly attenuated, and this effect was associated with a reduction in macrophage pro-inflammatory activity due to decreased IL-1β production. Mechanistically, UBE2M deficiency reduces VHL recruitment and destabilizes VHL via ubiquitination-mediated degradation. The resulting VHL loss alleviates HIF-1α degradation, leading to increased HIF-1α-induced IL-1β production [199]. Collectively, these findings establish neddylation-dependent macrophage activation as a key mechanism driving the pathogenesis of inflammatory diseases through NLRP3 [200,201,202]. 

#### 6.2.4. S-Palmitoylation

S-palmitoylation is catalyzed by palmitoyltransferases (PATs), also known as zinc finger DHHC-type (ZDHHC) proteins. Palmitoylation is closely related to inflammation. Intracellularly, palmitic acid-mediated modification of NLRP3 palmitoylation plays a key regulatory role in the assembly and activation of the NLRP3 inflammasome. At the onset of NLRP3 inflammasome activation, palmitoyltransferase ZDHHC5 palmitoylates NLRP3. This modification induces NLRP3 to bind tightly to NIMA-related kinase 7 (NEK7) during the subsequent assembly phase of activation, effectively stabilizing NLRP3 oligomers, which in turn promotes downstream caspase-1 activation, ultimately leading to the release of increased amounts of cytokines IL-1β and IL-18 to trigger an inflammatory response [203]. Researchers have demonstrated that treatment of macrophages with palmitoylation inhibitors such as 2-bromopalmitate (2-BP) or fatty acid synthase (FASN) inhibitors like cerulenin significantly suppresses pyroptosis induced by LPS/Nigericin. Further investigation reveals that 2-BP affect GSDMD-NT membrane trans-location but does not affect full-length GSDMD, thereby inhibiting pyroptosis and IL-1β release. Thus, palmitoylation of GSDMD-NT serves as a critical checkpoint for its membrane localization and subsequent activation [204]. Additionally, this study indicates that targeting the palmitoyltransferase zinc finger DHHC-type palmitoyl transferase 7 (ZDHHC7) or the NLRP3 cysteine residue at position 126 effectively attenuates NLRP3 inflammasome-mediated inflammatory responses [205]. The substrate palmitate is derived from CD36-mediated uptake or FASN-dependent de novo synthesis—both of which are enhanced in the obese adipose milieu and are known to supply palmitate for protein S-palmitoylation [206]. This directly couples local lipid availability to inflammasome licensing. Additionally, palmitoylation of TLR4, which senses signals from palmitic acid and LPS, enhances its partitioning into lipid rafts, affecting signalosome formation [207] (Table 2).

## 7. Therapeutic Directions on ATMs 

Understanding the metabolic reprogramming of ATMs has opened multiple avenues for therapeutic intervention in obesity and associated metabolic disorders. Several targetable pathways have emerged from preclinical studies. CAMKK2 has been identified as a key metabolic checkpoint in pro-inflammatory macrophages; myeloid-specific ablation of CAMKK2 in mice reprograms macrophage metabolism toward an anti-inflammatory oxidative phenotype, enhances energy expenditure, and protects against diet-induced obesity and insulin resistance, positioning CAMKK2 as a promising kinase target [208]. PPARα/γ agonism selectively within ATMs represents another strategy: dextran-based nanomedicine delivering PPAR agonists to ATMs improved glucose tolerance within two weeks, promoted adipose tissue browning, and ameliorated hepatic steatosis in multiple rodent obesity models [209]. Restoration of protective ATM functions is also achievable via cytokine modulation; ATM-derived IL-10 suppresses hepatic gluconeogenesis and rescues hyperglycemia in obese mice, suggesting IL-10–producing ATMs restoration as a metabolically beneficial intervention [210]. Furthermore, targeting the Trem2-DAP12-SYK pathway with natural compounds, such as EGCG and SMRR, reactivates this LAM program and mitigates obesity in vivo, highlighting Trem2^+^ LAMs as an actionable subset [211]. Despite these advances, therapeutic modulation of ATM metabolism entails inherent risks that warrant careful consideration. ATMs are not uniformly pathogenic; specific subsets, particularly Trem2^+^ LAMs, play dual roles, underscoring the importance of subset-specific rather than global ATM targeting [6]. The spatial and depot-specific heterogeneity of ATMs further complicates therapeutic design, as visceral versus subcutaneous ATMs and those localized in CLS versus interstitial spaces exhibit distinct metabolic and functional profiles. These considerations argue for strategies that reprogram ATM metabolism toward protective states rather than indiscriminate depletion or broad immunosuppression. Emerging technologies, including nanomedicine-enabled targeted delivery and subset-specific transcriptional modulators, offer routes to achieve precision targeting. Ultimately, the therapeutic potential of ATMs metabolic reprogramming lies in the ability to shift the balance from maladaptive inflammation toward adaptive tissue remodeling, a goal that will require deeper mechanistic understanding of ATMs subset heterogeneity and its integration with whole-body energy metabolism.

## 8. Conclusions

The intricate crosstalk between immunity and metabolism is pivotal in the pathogenesis of obesity and diabetes. This review systematically delineates the multifaceted roles of ATMs in this context. We examine how distinct macrophage subtypes, particularly MMe, contribute to adipose tissue homeostasis and dysfunction in obesity. Furthermore, we dissect the mechanisms underlying macrophage metabolic reprogramming, focusing on key transcription factors and PTM. Collectively, these insights establish a foundation for novel therapeutic strategies that target immunometabolic pathways in macrophages to combat obesity and its related metabolic diseases.

## Figures and Tables

**Table 1:** Phenotypes and functions of ATMs subsets.

Types	Activation Environment	Stimulus or Metabolic Stress	Functions	Refs
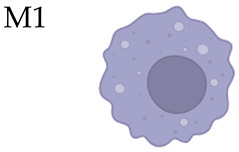	Inflammatory response and immune defense	LPS, IFN-γ, TNF-α, IL-1β, GM-CSF, IL-6	Anti-microbial, pro-inflammatory, anti-tumor, promote IR, and T2D	[31]
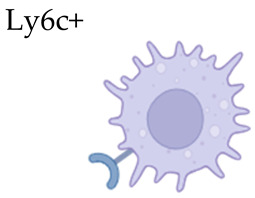	Metabolic changes and the inflammatory environment of adipose tissue under obesity	Inflammatory cytokine (TNF-α, IL-6, Leptin, Adiponectin)	Promoting inflammatory responses and metabolic disorders	[32]
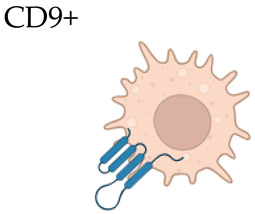	Hypoxia, IR, inflammatory environment, obesity	CCL2, IL-1α, IL-18, TNF	Phagocytosis and degradation of adipocytes (phagocytosis of fat droplets), inflammatory phenotype	[30]
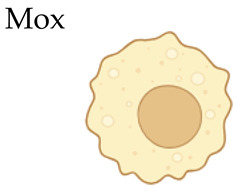	Oxidative tissue damage, obesity-induced IR	Oxidized lipids, LDL, ROS, Nrf2	Oxidative stress buffering, atherosclerotic lesion, redox-regulatory genes over-representation	[33,34]
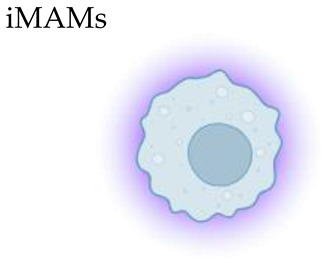	Chronic low-grade inflammation, disturbance of lipid metabolism, induce proinsulin misfolding and endoplasmic reticulum	PDIA3, ROS, FFA, Palmitic acid, TNF-α, MCP-1, IL-6	Pro-inflammatory, metabolic disorder, promote IR, lead to fat	[3]
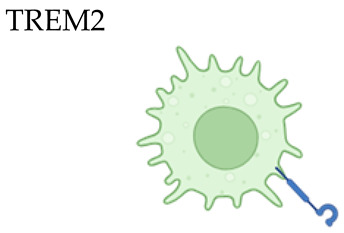	Tissue damage, metabolic dysregulation, neurological disorders and the tumor microenvironment	LDL, APOE, TNF-α, IL-6, M-CSF, amyloid, SIP, Obesity	Regulation of macrophage differentiation and survival, phagocytosis and lipid metabolism functions, regulation of metabolic syndrome	[6,35,36]
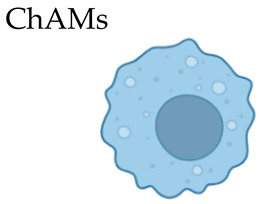	Cold or excess calorie intake, diet-induced thermogenesis	CHRNA2, Ach, GABPα	Induce adaptive thermogenesis, express crucial genes for ACh synthesis and secretion in mice	[10,37,38]
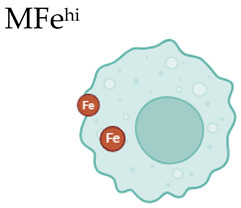	Iron overload	CD163, Tfrc, Fth1, Ftl1, Slc40a1, Hmox1, Cp	Iron recycling	[39]
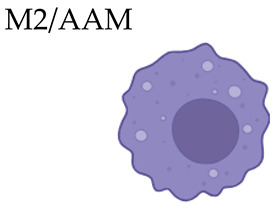	Tumor microenvironment, wound healing, inflammatory state, high glucose environment	IL-4, IL-10, IL13, Glucocorticosteroid	Anti-inflammatory, tissue repair and remodeling, immunomodulation, promoting the formation and function of beige fat	[40]
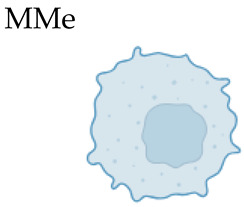	Obese adipose tissue, hypoxic and inflammatory microenvironment, lipid-rich and necrotic adipocyte surroundings	Saturated fatty acids	Pro-inflammatory cytokine secretion, lipid buffering and clearance	[41]

Macrophages exhibit remarkable plasticity, and their functional phenotypes are dynamically in response to environments. The biological effects and stimulus of macrophages are shown. CHRNA2, Cholinergic Receptor Nicotinic Alpha 2 Subunit. ChAMs, Cholinergic Adipose Macrophages. Insulin resistance, IR.

**Table 2:** Post-translational modifications in the regulation of inflammation.

PTM Types	Enzymes	Target Proteins/Substrates	Functional Consequences /Inflammatory Outcomes	Refs
Acetylation	ACLY	Histone H3K27	↑ Inflammation	[177]
	p300/CBP	Histones	↑ Inflammation resolution	[180,190]
Deacetylation	HDAC3	HADHA	↓ FAO ↑ IL-1β production	[183]
		NF-κB p65 (Lys122)	↑ Inflammation and fibrosis	[184]
		—	↑ Anti-inflammatory effects	[189]
Lactylation	—	Histone H3/Histone Kla	↑ Inflammation resolution	[141]
	—	HMGB1	↑ Inflammation	[191]
O-GlcNAcylation	OGT	S6K1 (S6 kinase beta-1)	↓ Inflammation ↑ Healthy adipose tissue expansion	[194]
Neddylation	Ubc12/UBE2M	TRIM21 on K129/134	↑ Inflammation ↑ Obesity ↑ Insulin resistance	[199]
S-palmitoylation	ZDHHC5	NLRP3	↑ Inflammation	[203]
	ZDHHC7	NLRP3 (Cys126)	↓ Inflammation	[205]

## Data Availability

No new data were created or analyzed in this study.
